# Laser-Based Additive Manufacturing Processability and Mechanical Properties of Al-Cu 224 Alloys with TiB Grain Refiner Additions

**DOI:** 10.3390/ma18030516

**Published:** 2025-01-23

**Authors:** Esmaeil Pourkhorshid, Paul Rometsch, X.-Grant Chen

**Affiliations:** 1 Department of Applied Science, University of Quebec at Chicoutimi, Saguenay, QC G7H 2B1, Canada; epourkhors@etu.uqac.ca; 2 Arvida Research and Development Center, Rio Tinto Aluminium, Saguenay, QC G7S 4K8, Canada; paul.rometsch@riotinto.com

**Keywords:** Al-Cu 224 alloy, selective laser melting, TiB grain refiner, hot tearing susceptibility, microstructural characterization, mechanical properties

## Abstract

This study investigated the impact of TiB grain refiner additions on the microstructural evolution, hot tearing susceptibility, and mechanical properties of Al-Cu 224 alloys to enhance their processing performance during the selective laser melting (SLM) process. A simple laser surface remelting method was utilized to simulate laser-based rapid solidification. The results revealed that the addition of appropriate amounts of TiB grain refiner could completely eliminate the solidification cracks during the laser surface remelting process. The introduction of TiB_2_ particles in the melt pools through the TiB grain refiner addition changed the grain morphology from a coarse columnar to a fine equiaxed structure, and the grain sizes were reduced from 13 to 15 μm in the base alloys to 5.5 μm and 3.2 μm in the alloys with 0.34 wt% Ti (B-3TiB) and 0.65 wt% Ti (ZV-6TiB) additions, respectively. The hardness values of the modified B-3TiB and ZV-6TiB alloys reached 117 and 130 HV after a T6 heat treatment, which surpassed the hardness of conventional AlSi10Mg alloys by at least 15–30%. This improvement was attributed to the finer grains and nanoscale θ′/θ″ precipitates. The results demonstrate that the TiB grain refiner addition can significantly improve the processability and mechanical properties of Al-Cu 224 alloys for SLM applications, offering a promising solution to the challenge of high hot tearing susceptibility in high-strength aluminum alloys.

## 1. Introduction

The demand for high-strength aluminum alloys has significantly increased recently, driven by advancements in high-tech industries such as aerospace, automotive, and electronics [1,2]. Among these alloys, Al-Cu alloys, including typical 206 and 224 cast alloys and AA2024 and AA2219 wrought alloys, are able to achieve tensile strengths of 400–500 MPa [3,4,5]. This is primarily due to the presence of numerous nanoscale precipitates (Al_2_Cu and S-Al_2_CuMg), which form after solutionizing and subsequent artificial aging and thereby provide the main strengthening effect [6]. The precipitation strengthening can be further enhanced by adding transition alloying elements, such as Zr, V, Ti and Sc. It has been reported that microalloying with Zr, V and Ti in Al-Cu 224 cast alloys can significantly improve the mechanical properties at both room and elevated temperatures [7,8,9], thereby enhancing their industrial applicability for various components in the internal combustion engines, heat-resistant materials, and fire-resistant structures.

Selective laser melting (SLM), a powerful additive manufacturing process, has gained a great deal of attention in recent years owing to its ability to create complex-shaped materials [10]. For example, some components of vehicle engines require high strength at both room and elevated temperatures and can be efficiently built by SLM, thereby reducing production time and increasing efficiency [11]. However, the rapid solidification nature of the SLM process introduces challenges such as residual stresses and a high risk of solidification cracks [10,12,13]. Consequently, the production of aluminum products by SLM has primarily been limited to alloys that are highly castable or weldable. Although high-Si aluminum alloys, such as typical AlSi10Mg, are good candidates for SLM, their mechanical properties are much lower than those of high-strength aluminum alloys, such as 2xxx, 6xxx, and 7xxx alloys [14]. However, high-strength aluminum alloys are generally susceptible to severe hot tearing during SLM, which limits their widespread use [15].

Controlling hot tearing in high-strength aluminum alloys during SLM has emerged as a critical area of research. The research can be broadly divided into two main approaches: optimizing the SLM process parameters [16,17] and modifying the chemical composition of alloys [13,18,19]. While the former requires a higher energy density, which usually reduces the production rate, the latter can effectively modify the solidification and control cracking. Preheating the powder during the SLM process has demonstrated certain advantages in enhancing processability. For instance, Uddin et al. [20] utilized powder bed preheating up to 500 °C to achieve crack-free fabrication of the alloy AA6061. However, using high-temperature heating in SLM alone is not an efficient method owing to high energy consumption. Tan et al. [21] reported that the processing parameter window of alloy AA2024 for the lowest amount of hot cracking produced the highest porosity fraction and the lowest sample density. In recent years, chemical composition modification has been widely used. Benoit et al. [18] studied the effect of minor Fe additions on the crack susceptibility of alloy AA6060 and reported that the solidification cracks of AA6060 could be controlled with increasing Fe content of up to 0.5 wt%. The co-incorporation effect of Si and TiB_2_ for increasing the fracture toughness to resist hot cracking in the 7xxx alloys was studied by Zhou et al. [22]. While Si added extra fluidity to the alloy and reduced solidification shrinkage, TiB_2_ remained stable during SLM and functioned as a nucleating agent. Zheng et al. [23] recently examined the effect of TiC nanoparticles in 7075 alloy during SLM. Their results showed that the internally dispersed TiC nanoparticles in 7075 powders successfully eliminated hot cracking during the SLM process. Sun et al. [19] investigated Er- and Zr-modified Al-Mg alloys after SLM and reported that the good processability of the modified alloys was associated with the grain-refining effect of Er and Zr. A Ti-modified Al-Cu-Mg alloy was developed specifically for SLM by Zhang et al. [24]. The results indicated that the Ti addition can effectively promote the transition from columnar to equiaxed grains in the modified alloy owing to the heterogeneous nucleation effect of Al_3_Ti. It was also reported that a 2 wt% Zr addition to the base Al-Cu-Mg alloy prevented the formation and propagation of cracks during SLM [25].

Although some previous works have involved modifying the Al-Cu alloys for the SLM process [21,26,27], full control over hot tearing in high-strength Al-Cu alloys has yet to be achieved. In addition, the feasibility of utilizing commercial Al-Cu 224 alloy in SLM has not been reported in the literature. This study aims to demonstrate an approach for eliminating solidification cracks in rapidly solidified 224 alloy through the addition of appropriate amounts of TiB grain refiner. Using TiB grain refiner, a widely utilized master alloy in the aluminum industry, and preparing the powders without additional mechanical alloying step to form TiB_2_ particles can significantly reduce production costs. The effect of the TiB grain refiner on the performance of the SLM process was investigated using a laser surface remelting method [28,29,30], which simulated the rapid solidification conditions, especially during laser-based manufacturing processes. Five Al-Cu 224 alloys with and without added TiB grain refiner were prepared by casting and laser surface remelting techniques to compare the hot tearing susceptibility, microstructure, and mechanical properties. This study provides a new approach and insights into enhancing the hot tearing resistance and mechanical properties of high-strength Al-Cu 224 alloys for SLM applications.

## 2. Materials and Methods

### 2.1. Materials Preparation

Five Al-4.5Cu based alloys were prepared. The first alloy was a typical Al-Cu 224 cast alloy containing 4.7% Cu and 0.35% Mn with minor Mg and Fe additions (0.10–0.12% for each), and is denoted as the base alloy. The second alloy was modified with a 0.34% Ti addition to the base alloy using Al-5Ti-1B master alloy and is denoted as B-3TiB alloy. The third alloy includes 0.15% Zr and 0.20% V additions to the base alloy and is designated as the ZV alloy. The fourth and fifth alloys were modified with 0.3% and 0.65% Ti additions using Al-5Ti-1B master alloy to the third alloy, and are designated as ZV-3TiB and ZV-6TiB alloys, respectively. Ti concentrations were chosen based on preliminary results and cost-effectiveness, ensuring effective control of hot tearing. All alloys were placed in an electric resistance furnace with a melting temperature of 750 °C and then poured into a copper permanent mold to obtain thin-wall cast plates measuring 110 mm × 110 mm × 4 mm with a solidification cooling rate of ~20 °C/s. The chemical compositions of five alloys analyzed using optical emission spectroscopy are listed in Table 1. All chemical compositions in this study are given in wt% unless otherwise indicated. Samples with dimensions of 40 mm× 10 mm× 4 mm were machined from the cast plates, sand-blasted, and then ground using SiC paper to maintain the same surface condition.

**Figure 1:**
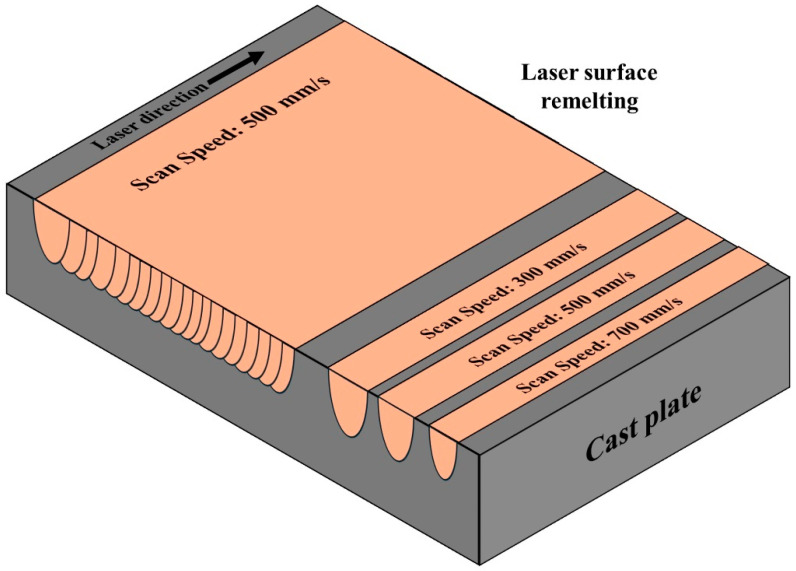
Schematic of the laser surface remelting sample and process.

These samples were placed on the substrate of an SLM SOLUTIONS 125 machine (SLM Solutions Group AG, Lübeck, Germany) equipped with a 400 W IPG laser for laser surface remelting (LSR) under an Ar atmosphere. During the LSR process, linear remelting tracks were produced with a laser power of 370 W, a spot size of 100 μm, and scan speeds of 300, 500, and 700 mm/s. In addition, 20 parallel line scans with the same laser power and spot size, a scan speed of 500 mm/s and a hatch distance of 130 µm were performed for each alloy to generate a region of overlapping melt pools for hardness measurement. This approach was used to explore the effects of the process parameters and alloy compositions on the processability of Al-Cu alloys under the conditions that are similar to rapid solidification processing via SLM. A schematic of the LSR sample and process is shown in Figure 1.

**Figure 2:**
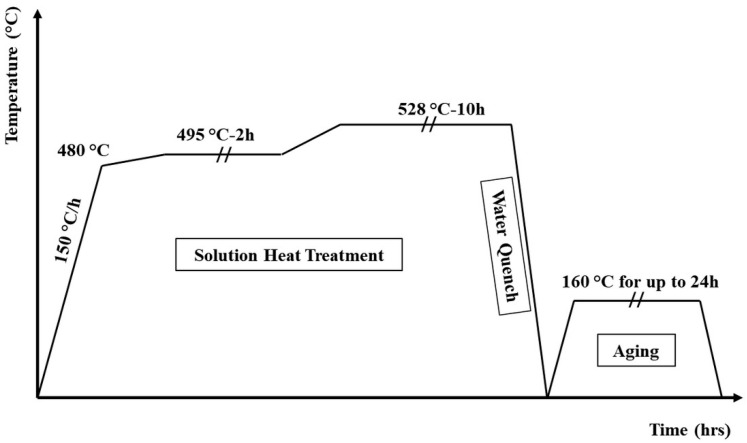
Schematic of the T6 heat treatment applied in this study.

To study the aging response and the potential of precipitation strengthening, a portion of the LSR samples were subjected to a T6 heat treatment, which included a two-step solution treatment at 495–528 °C followed by water quenching, and then artificial aging at 160 °C for different lengths of time. The heat treatment procedure is illustrated in Figure 2.

### 2.2. Microstructural and Mechanical Characterization

Transverse cross-sections of the laser-melted linear tracks were prepared by a standard metallographic procedure for microstructural analysis. At least five cross-sections along the length of the tracks were investigated for each alloy composition and process condition. An optical microscope and a scanning electron microscope (SEM, JEOL JSM-6480LV, JEOL USA Inc., Peabody, MA, USA) were used to examine the melt pool microstructures. The grain structures were characterized using the electron backscatter diffraction (EBSD) technique. The EBSD data were collected using an accelerating voltage of 20 kV and a step size of 1 μm and processed using the HKL Channel 5 software (version 5.0.9.0). A transmission electron microscope (TEM, JEOL JEM-2100, JEOL USA Inc., Peabody, MA, USA) was also used to investigate the precipitation microstructures inside the pool after the T6 heat treatment. The TEM samples were prepared in a twin-jet polishing unit operated at 20 V in a solution consisting of 30 vol% nitric acid and 70 vol% methanol at −30 °C after the grinding and polishing procedure. The thicknesses of the TEM samples were measured using a convergent beam diffraction pattern. The mechanical properties of each pool were evaluated based on the Vickers hardness using an NG-1000 CCD machine (NextGen Material Testing, Vancouver, BC, Canada). The hardness measurements were performed at a load of 25 g and a dwell time of 20 s. At least ten indentations were made to obtain the average value in the parallel scan areas.

### 2.3. Solidification Simulation and Hot Tearing Index

ThermoCalc software (version 2023 with the TCAL7 database) was used to simulate the solidification path of the alloys. The Scheil–Gulliver (non-equilibrium) solidification mode was used for the simulation, with a maximum temperature step size of 1 °C. The simulation predicted the temperature (T) versus solid fraction (f_s_) curve and the formation of intermetallic phases. The hot tearing susceptibility of the studied alloys was accessed using Easton’s criterion with the following equation [31]:

(1) $${\mathrm{HTI}}_{E}=\int_{T_{0}}^{T_{\mathrm{CO}}}f_{s}\left(T\right)\cdot \mathrm{dT}$$

In this method, two parameters are considered: T_0_, denoting the temperature of coherency, where feeding transitions interdendritically, and T_CO_, the temperature of coalescence, where solidification reaches a point of solidity. In this study, T_0_ was set at 0.7 and T_CO_ was set at 0.98.

## 3. Results

### 3.1. As-Cast Microstructures

**Figure 3:**
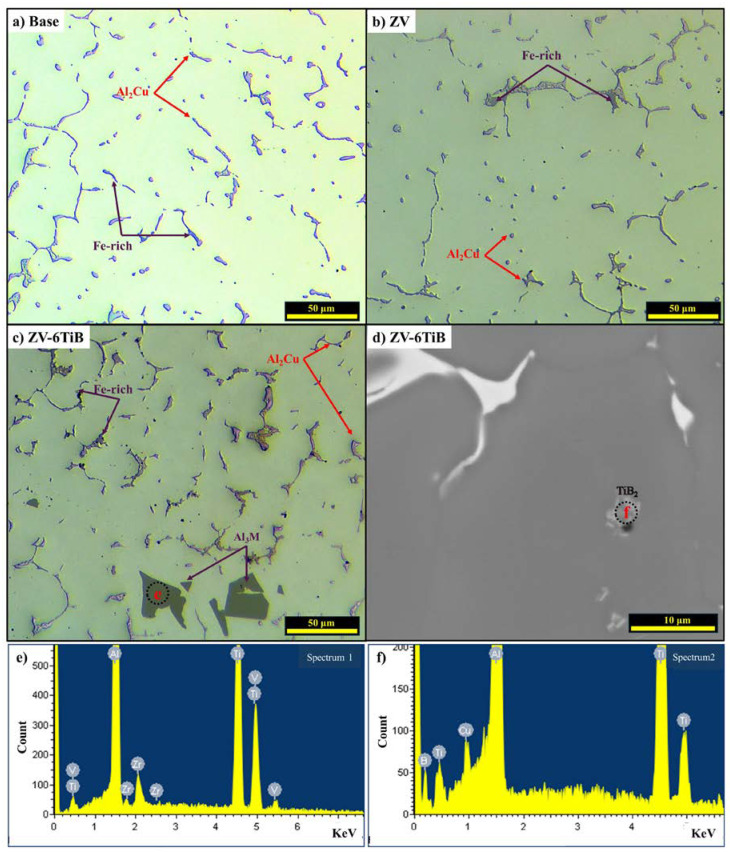
As-cast optical microstructures: (**a**) base, (**b**) ZV, and (**c**) ZV-6TiB alloys, (**d**) an enlarged SEM backscattered image from ZV-6TiB alloy showing fine TiB_2_ particles, (**e**) SEM-EDS spectrum of Al_3_Ti-type intermetallic particles in (**c**), (**f**) SEM-EDS spectrum of TiB_2_ particles in (**d**).

The as-cast microstructures of the base and ZV alloys are shown in Figure 3a,b. Both alloys exhibited similar microstructures, which consisted of Al grains with Al_2_Cu and Fe-rich intermetallics distributed along the cell/grain boundaries. The Fe-rich intermetallics were mainly composed of Al_7_Cu_2_(Fe, Mn) and Al(Fe, Mn)Si, determined by the phase morphology and SEM-EDS analyses, and relevant literature [7,9]. The majority of the Zr and V in the ZV alloy was dissolved in the matrix, which accounts for the scarcity of Zr/V-containing intermetallics found in the matrix [7]. With the addition of TiB grain refiner in both alloys, the most notable changes were the presence of Ti-containing intermetallics and TiB_2_ particles in the matrix, as exemplified in the ZV-6TiB alloy (Figure 3c,d). The excess Ti produced Al_3_Ti-type intermetallic particles, and the SEM-EDS analysis revealed that some Zr and V dissolved in the Al_3_Ti-type intermetallic particles (Figure 3e). The average size of those particles is 10 μm. An enlarged SEM image of ZV-6TiB alloy showed some fine particles with an average size of 1 μm; these were mostly distributed in the middle of the Al grains (Figure 3d). The SEM-EDS analysis indicated that these were TiB_2_ particles (Figure 3e).

### 3.2. Microstructures After Laser Surface Remelting

#### 3.2.1. Cracking in the Melt Pools

**Figure 4:**
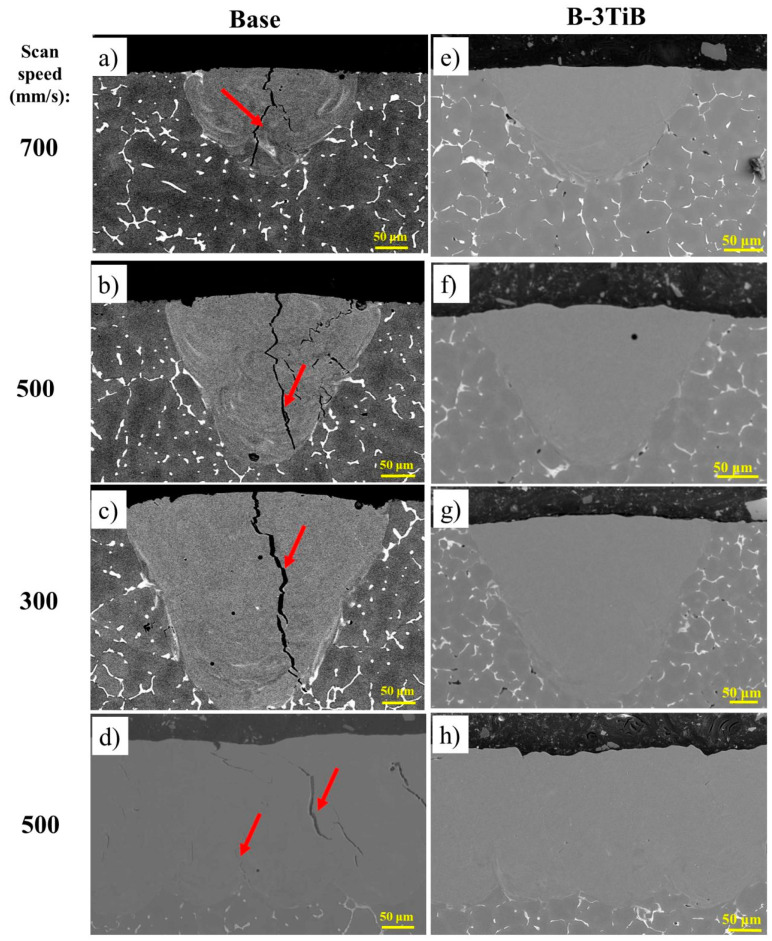
SEM micrographs showing cross-sections of melt pools at different laser scan speeds: (**a**–**d**) for the base alloy, with cracks indicated by red arrows; and (**e**–**h**) for the B-3TiB alloy.

The melt pools after LSR for the base and B-3TiB alloys are shown in Figure 4. In the base alloy, macro cracks can be seen in all melt pools regardless of the laser scan speed (a–d). These cracks, indicated by red arrows, were principally located in the middle of the melt pool and localized between adjacent grains. It was reported that such cracks occur during the last stages of solidification, where coherency in the solidifying melt pool begins [18]. The large solidification interval of Al-Cu alloys was identified as the main reason for the high cracking susceptibility in the final stage of solidification [16], where the liquid phase cannot fully penetrate between the grains, and the thermal contraction of the solidified material causes high stresses that exacerbate cracking [32]. In contrast, the melt pools of the B-3TiB alloy were solidified completely without any cracks (Figure 4e–h). This indicates that the incorporation of the TiB grain refiner can effectively control cracking in the melt pools.

**Figure 5:**
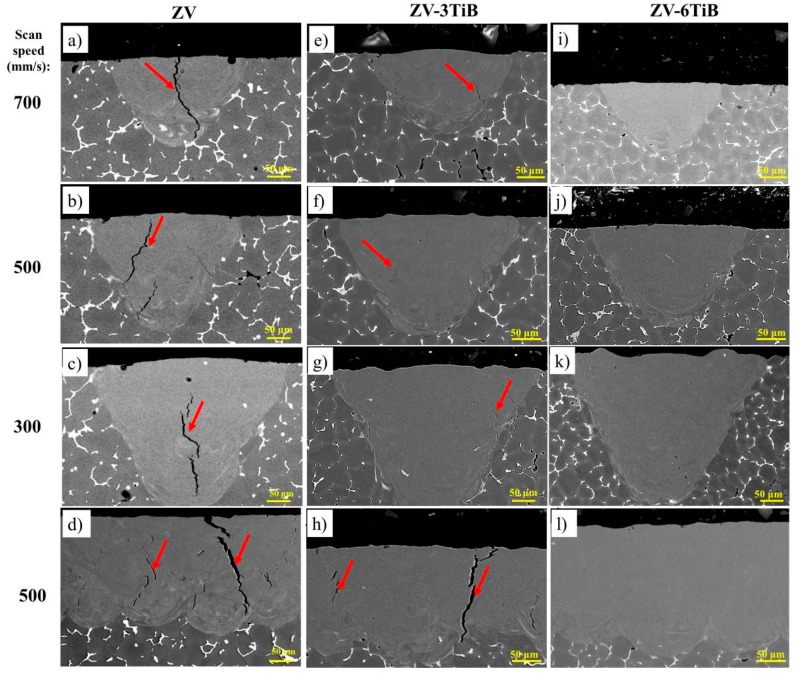
SEM micrographs showing cross-sections of melt pools under different laser scan conditions: (**a**–**d**) for the ZV alloy, (**e**–**h**) for the ZV-3TiB alloy, and (**i**–**l**) for the ZV-6TiB alloys. The cracks are indicated by red arrows.

The melt pools for the ZV alloy and two other modified alloys are presented in Figure 5. In the ZV alloy (Figure 5a–d), severe macro cracks occurred in all melt pools regardless of the scan speeds used in the LSR process. In the ZV-3TiB alloy (Figure 5e–h), some light cracks were observed in the single-scan melt pools at different speeds, but severe cracks could still be observed in the multilayer area scanned at 500 mm/s. Compared with the cracks in the ZV alloy, the number and severity of cracks in the melt pools decreased. While the cracking in the B-3TiB alloy could be controlled with the addition of 0.3%Ti by Al-5Ti-1B grain refiner addition, the same amount of grain refiner in the ZV-3TiB alloy could not prevent some cracks from appearing in the melt pools. This clearly demonstrates a higher tendency for cracking in the ZV alloy than in the base alloy. Following further modification by a higher amount of grain refiner addition in the ZV-6TiB alloy (Figure 5i–l), all cracks disappeared in the melt pools, whether in single tracks with different scan speeds or in multilayer areas.

#### 3.2.2. Grain Sizes in the Melt Pools

**Figure 6:**
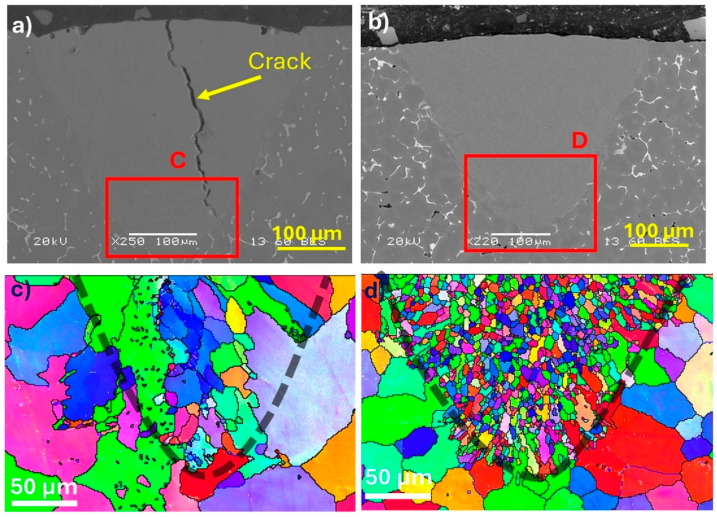
SEM micrographs of the melt pools at a 300 mm/s scan speed for (**a**) the base alloy and (**b**) the B-3TiB alloy; (**c**) EBSD orientation map of the base alloy from the selected area C of (**a**), and (**d**) EBSD orientation map of the B-3TiB alloy from the selected area D of (**b**). The dotted gray lines of (**c**,**d**) indicate the melt pool boundaries (The different colors in (**c**,**d**) indicate distinct grain orientations).

**Figure 7:**
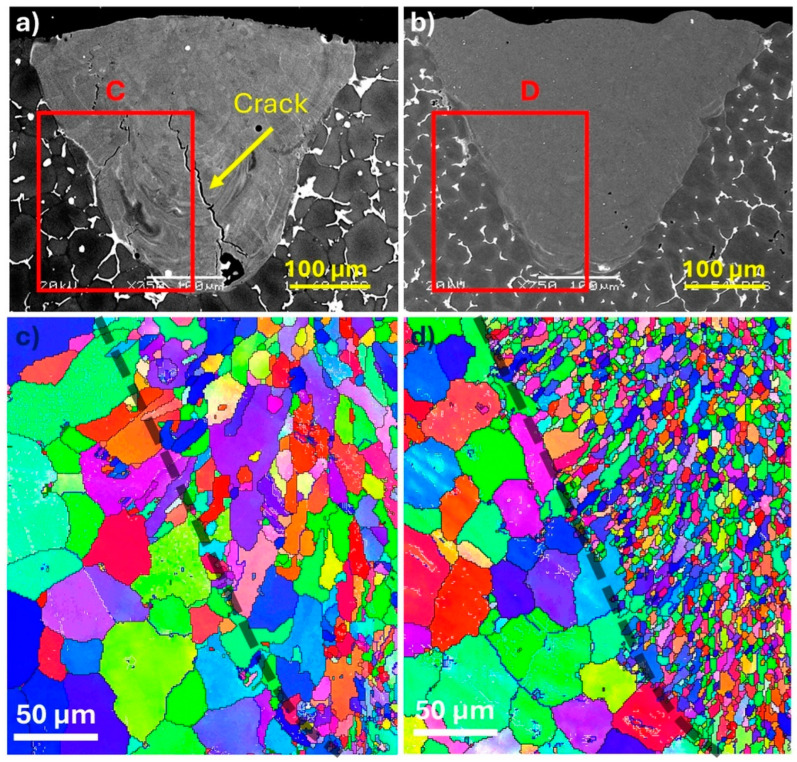
SEM micrographs of the melt pools at a 300 mm/s scan speed for (**a**) the ZV alloy and (**b**) the ZV-6TiB alloy; (**c**) EBSD orientation map of the ZV alloy from the selected area C in (**a**), and (**d**) EBSD orientation map of the ZV-6TiB alloy from the selected area D in (**b**). The dotted gray lines of (**c**,**d**) indicate the melt pool boundaries (The different colors in (**c**,**d**) indicate distinct grain orientations).

In conventional casting, grain refinement can be achieved through inoculation treatments that add potent nucleation particles or suitable solutes to inhibit grain growth [33]. In contrast, the high cooling rate of LSR reduces constitutional supercooling effects, even where a high level of Cu acting as the main solute in the base and ZV alloys, thereby causing large elongated grains to form due to epitaxial growth (Figure 6c and Figure 7c). Therefore, researchers have adopted inoculation treatments using nucleation particles such as Al_3_Ti [34] and Al_3_Zr [25], or adding Er and Zr [19] in SLMed aluminum alloys to control the grain structure.

The melt pools in the base and B-3TiB alloys are shown in Figure 6a and b, respectively. The EBSD orientation map of the base alloy shows that most grains in the melt pool were large and elongated in the heat transfer direction (Figure 6c). These grains were grown epitaxially from the partially melted substrate at the melt pool boundary, as observed in the literature [35]. The average grain size in the base alloy was measured to be 13.4 μm.

In contrast, the EBSD orientation map of the modified B-3TiB alloy shows mostly equiaxed and smaller grains with an average grain size of 5.5 μm (Figure 6d). The incorporation of the TiB grain refiner significantly reduced the grain size in the melt pools. Similar effects were reported in that TiC nanoparticles promoted the columnar-to-equiaxed grain transition in SLMed AA2024 Al alloys [35]. Martin et al. reported [13] that ZrH_2_ pre-alloyed 7075 and 6061 Al powders increased nucleation sites, leading to a fully equiaxed grain structure in SLM-processed alloys. It was also observed that microscale Al_3_(Sc,Zr) precipitates facilitated a fully equiaxed grain structure in an Al-Mg-Sc-Zr alloy produced via direct energy deposition [36].

Figure 7a,b show the melt pools for the ZV and ZV-6TiB alloys, respectively. The EBSD orientation map of the ZV alloy revealed that most grains were mainly elongated in the heat transfer direction (Figure 7c). In contrast, the ZV-6TiB alloy exhibited a fine, equiaxed grain structure. The average grain sizes were reduced from 15.4 μm in the ZV alloy to 3.2 μm in the modified ZV-6TiB alloy. These results also show that the higher level of grain refiner addition in the ZV-6TiB alloy reduced the grain size more effectively than that in the B-3TiB alloy.

**Figure 8:**
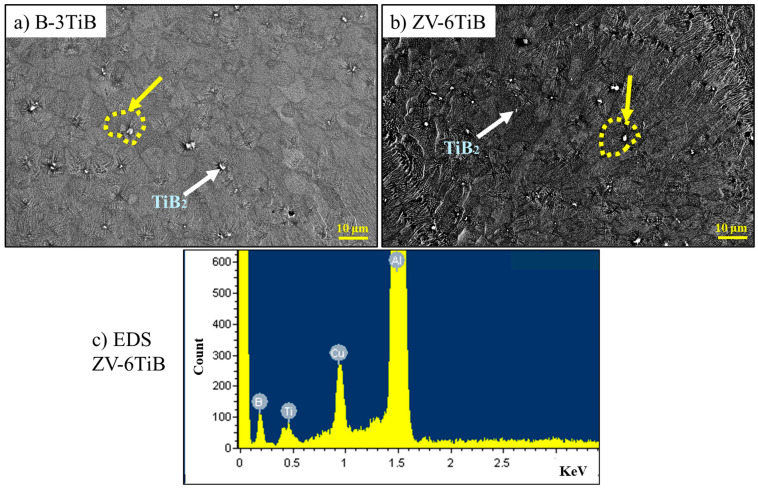
Microstructures in the centers of melt pools (300 mm/s) for (**a**) the B-3TiB alloy and (**b**) the ZV-6TiB alloy; (**c**) SEM-EDS result of a small TiB2 particle in (**b**). The dotted yellow circles indicate the grain boundaries.

Figure 8 shows the microstructures of the B-3TiB and ZV-6TiB alloys in the centers of melt pools. In both alloys, α-Al solidified with cellular structures, while Cu-rich intermetallics solidified in the intercellular regions. Both alloys exhibit small equiaxed grains, as demonstrated in Figure 6d and Figure 7d. Small particles can be observed in the middle of the majority of the grains, and they appear as white spots in the backscatter SEM images (as indicated by the yellow circles in Figure 8a,b). The EDS analysis of those particles in both alloys indicates that they are TiB_2_ particles (Figure 8c). The average size of the TiB_2_ particles is ~0.9 μm in both alloys. The number densities of the TiB_2_ particles in the B-3TiB and ZV-6TiB alloys are 2.8 × 10^−3^ and 5.6 × 10^−3^ μm^−2^, respectively. In general, TiB_2_ particles have excellent compatibility with the aluminum matrix owing to their good wettability with Al liquid and their excellent properties such as their high hardness, high stiffness, high melting point and considerable chemical stability. It is reported that the TiB_2_ particle is an excellent grain growth inhibitor in the Al matrix, which can provide low-energy-barrier heterogeneous nucleation sites ahead of the solidification front and can induce a change in the grain morphology, causing it to evolve from a columnar to a fine equiaxed structure [13].

### 3.3. Effect of T6 Heat Treatment

#### 3.3.1. Mechanical Properties

**Figure 9:**
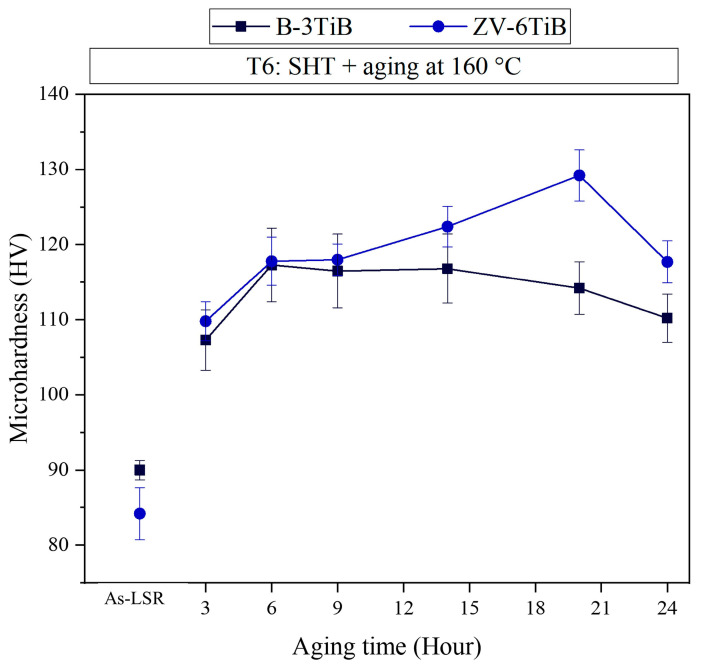
Aging response of the B-3TiB and ZV-6TiB alloys during T6 aging treatment.

To achieve the maximum mechanical properties of the two TiB-modified alloys (B-3TiB and ZV-6TiB), they were subjected to a T6 heat treatment, including a solution treatment followed by water quenching, and then artificial aging at 160 °C (Figure 2). The microhardnesses of both alloys as a function of aging times are shown in Figure 9. The initial hardness of tested samples before the LSR process was approximately 75 HV, and then the hardness of two tested materials increased to 85–90 HV after the LSR process. After aging for 3 h, the hardness was improved to ~110 HV, which is attributed to precipitation strengthening in the T6 condition [7]. After more than 8 h of aging, the hardness of the ZV-6TiB sample was clearly higher than that of the B-3TiB sample. The B-3TiB alloy was already peak-aged after 6 h, while the ZV-6TiB alloy was only peak-aged after 20 h with a higher hardness of 130 HV, compared to 117 HV for the B-3TiB alloy in the peak-aged condition. The peak-aged B-3TiB and ZV-6TiB samples were selected for further microstructural characterization.

#### 3.3.2. Microstructural Evolution

**Figure 10:**
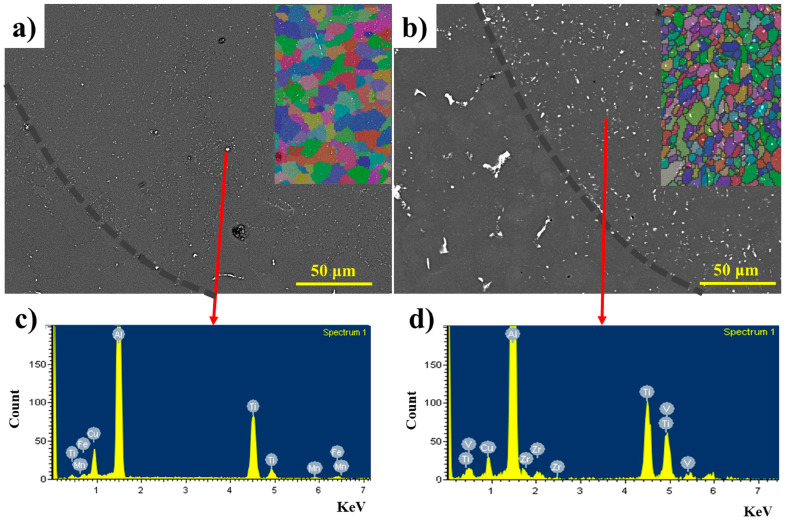
Microstructures of the melt pools after T6 heat treatment: (**a**) the B-3TiB alloy and (**b**) the ZV-6TiB alloy; (**c**) SEM-EDS result of the particles in (**a**); (**d**) SEM-EDS result of the particles in (**b**). (The different colors in subfigures (**a**,**b**) indicate distinct grain orientations).

The microstructures and grain structures in the melt pools of the B-3TiB and ZV-6TiB alloys after peak-aged T6 treatments are presented in Figure 10. Following T6 solution treatment, the cellular structures in both samples completely disappeared, although some intermetallic particles persist in the Al matrix. EBSD analysis indicates that the average grain size of the B-3TiB alloy slightly increased to 6.5 μm compared to the as-laser scanned condition, while the average grain size of the ZV-6TiB alloy remained approximately the same at 3.5 μm. SEM-EDS analysis of the intermetallic particles indicated that they are Ti-rich intermetallics (Figure 10c,d). This suggests that the high density of such intermetallic particles in the ZV-6TiB alloy plays a significant role in maintaining its small equiaxed grain size after the solution treatment in the T6 condition.

**Figure 11:**
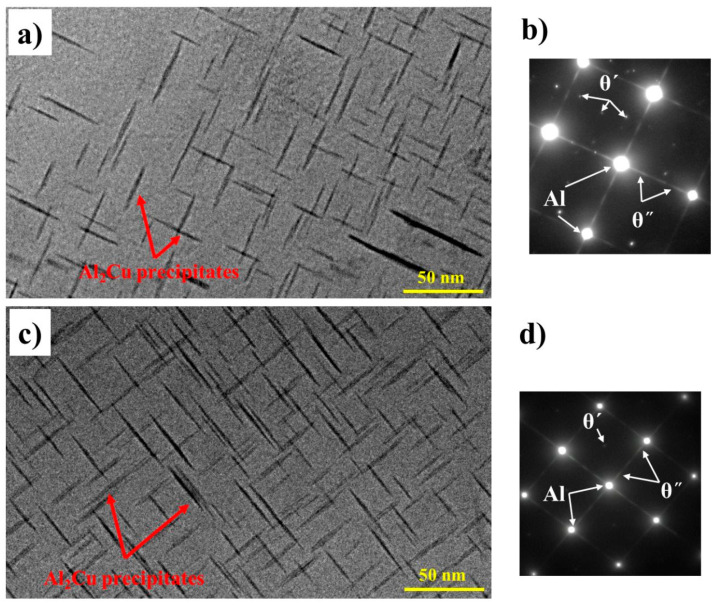
Bright-field TEM images viewed along <001>_α-Al_ showing the precipitation microstructures: (**a**) the B-3TiB alloy, and (**c**) the ZV-6TiB alloy in their respective peak-aged T6 conditions. The disk-shaped precipitates in the Al matrix were θ′- and θ″-Al_2_Cu precipitates, as indicated by the corresponding SADPs in (**b**) B-3TiB alloy, and (**d**) ZV-6TiB alloy.

The bright-field TEM images in Figure 11, viewed along the <001>_α-Al_ direction, show the precipitation microstructures of the B-3TiB and ZV-6TiB alloys after T6 aging treatment. Two types of disk-shaped Al_2_Cu precipitates were observed in the Al matrix. According to their size, shape, and the corresponding selected area diffraction patterns (SADP), these nanoscale precipitates were mainly observed to be in the θ′-phase, and to a lesser extent, the θ″-Al_2_Cu phase [7,8], which were the main strengthening phases in both alloys. The average length of the θ′/θ″-Al_2_Cu precipitates in the B-3TiB alloy was measured to be 42 nm, and the average thickness was 3 nm. On the other hand, the average length and thickness of these precipitates in the ZV-6TiB alloy were 40 and 2.8 nm, respectively. The number density, N_v_, was calculated based on the number of precipitates (N_1_+ N_2_) measured along the two explicit (001)_Al_ directions in the TEM images using the following equation:

(2) $$N_{V}=\left(N_{1}+N_{2}\right)\frac{\left(1+\frac{t+d_{t}}{2\;\sqrt{A_{s}}}\right)}{A_{s}\;(t+d_{t})}$$

where A_s_ is the area of the TEM images, t is the thickness of the TEM foil, and d_t_ is the effective width of the precipitates. The number densities of the θ′/θ″-Al_2_Cu precipitates in the B-3TiB and ZV-6TiB alloys were determined to be 12,100 and 16,000 μm^−3^, respectively. Owing to its higher number density of θ′/θ″-Al_2_Cu precipitates in the Al matrix, it is expected that the strength of the ZV-6TiB alloy is higher than that of the B-3TiB alloy, which is consistent with results in Figure 9.

## 4. Discussion

### 4.1. Effect of Energy Density on Melt Pools

**Figure 12:**
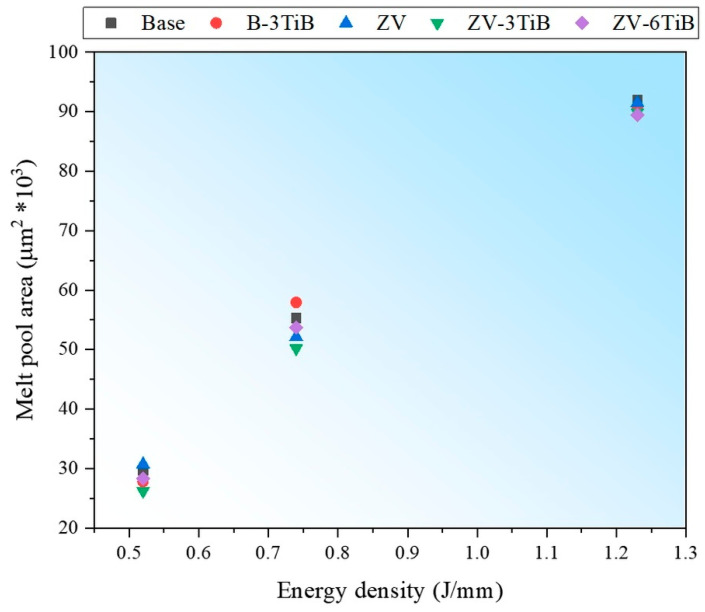
Effect of energy density on melt pool sizes for different alloys in single tracks of the LSR process.

Energy density is a critical parameter in materials processing, particularly in techniques such as laser welding and SLM manufacturing. It represents the amount of energy delivered per unit area and directly influences the characteristics of the melt pool, which is important for optimizing SLM processing to achieve the desired material properties. The linear energy density, ED_l_, is one of the most commonly used parameters in the literature, as shown in the following equation [10]:

(3) $${\mathrm{ED}}_{l}=\frac{P}{v}$$

where P is the laser power (W), and v is the scan speed (mm/s). This parameter is used in this study to compare the melt pool size in a single track of the LSR process. The effect of the linear energy density on the melt pool sizes of different alloys is shown in Figure 12. The presence of alloying elements and TiB_2_ particles in the studied alloys did not significantly affect the melt pool sizes at each energy density. However, as the linear energy density increased from 0.52 to 0.74 J/mm and further to 1.23 J/mm, the average melt pool areas increased by 88% and then by another 68%, respectively. Despite this, no relationship between energy density and cracking susceptibility was observed across all the samples. This is likely because of the similar range of input energy densities for the studied alloys, resulting in a comparable solidification rate under all conditions.

### 4.2. Hot Tearing Susceptibility

#### 4.2.1. ThermoCalc Calculation

**Figure 13:**
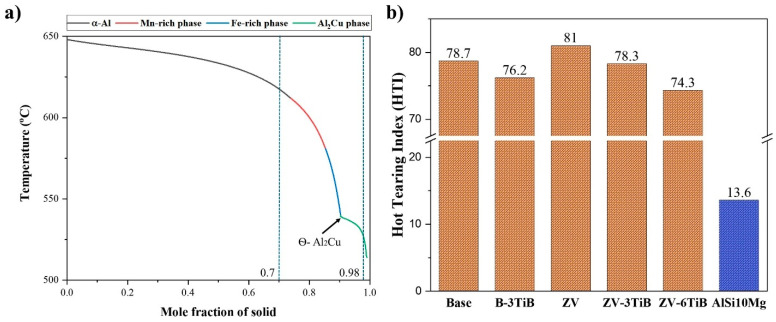
(**a**) Solidification sequence and temperature vs. solid fraction of the base 224 alloy predicted using the Scheil–Gulliver (non-equilibrium) mode in ThermoCalc, and (**b**) hot tearing susceptibility for the studied alloys predicted using Equation (1).

Figure 13a presents the solidification sequence of the base 224 alloy. The solidification began at approximately 650 °C with α-Al. The Mn-rich and Fe-rich intermetallic phases started to be precipitated around 612 °C and 580 °C, respectively. In the final stage of solidification, Al_2_Cu intermetallics emerged at ~530 °C, and solidification ended at 513 °C. The solidification interval of this alloy is considerably broad (approximately 140 °C). This is significantly broader than that of the commonly used AlSi10Mg alloys in SLM, which has a solidification interval of approximately 35 °C. The extensive interval in Al-Cu 224 alloys significantly increases the susceptibility to hot tearing during solidification. To numerically compare the hot tearing susceptibility for the studied alloys, Easton’s criterion using the hot tearing index, HTI_E_ in Equation (1), was applied [31]. For the alloys modified with TiB grain refiner, it was assumed that all B reacts with Ti to form TiB2 particles, and hence only excess Ti in the Al matrix was considered [37].

Figure 13b shows a comparison of the hot tearing index for the studied alloys. All alloys, including the modified ones, exhibited a higher HTI_E_ compared to the conventional AlSi10Mg alloys. The ZV alloy showed a higher HTI_E_ than the base alloy. With the addition of TiB grain refiner to modify the chemical composition, the HTI_E_ only slightly decreased in the modified alloys, and it remains significantly higher than that of the commonly castable AlSi10Mg alloys. The TiB additions in the B-3TiB and ZV-6TiB alloys completely eliminated the hot cracks in the laser surface remelting process and this corresponds to the lower HTI_E_ values for these alloys in Figure 13b. However, since these HTI_E_ values are only slightly lower than the others, this suggests that the modification of the chemical composition by excess Ti is not the primary factor for reducing the hot tearing susceptibility.

#### 4.2.2. Effect of the Grain Size

In general, thermal shrinkage, along with a broad solidification interval, is primarily responsible for the occurrence of hot tearing during solidification, especially in high-strength aluminum alloys such as the 2xxx/2xx and 7xxx/7xx series [13,38]. In this study, the base and ZV alloys have a solidification interval of ~140 °C, which can cause high shrinkage tension in the final stages of solidification. The linear thermal shrinkage can be described by the following equation:

(4) ε=α d ∆T

where ε is the thermal contraction in the solidified part, α is the coefficient of thermal contraction, d is the average grain size of the solidified part, and ΔT is the solidification interval. It is evident that larger grains produce higher shrinkage contraction in the final stages of solidification, leading to greater stress. This increased stress is applied to the liquid film between the grains. Additionally, surface tension exists within the liquid film between two adjacent grains during the final stage of solidification. This surface tension induces a compressive stress σ on the liquid film and can be expressed as follows [39]:

(5) $$\sigma =\frac{2\gamma }{b}$$

where γ is the surface tension and b is the thickness of the liquid film between two grains. When smaller grains are present, the grain boundary area significantly increases, and the liquid film thickness is smaller compared to that of bigger grains. According to Equation (5), the compressive stress acting on the liquid film between two grains is therefore lower with large grains. When the tensile stress caused by thermal contraction is larger than the compressive stress, cracking will occur in the liquid film between two grains. Therefore, cracks are more likely to occur within the coarse-grained structure.

**Figure 14:**
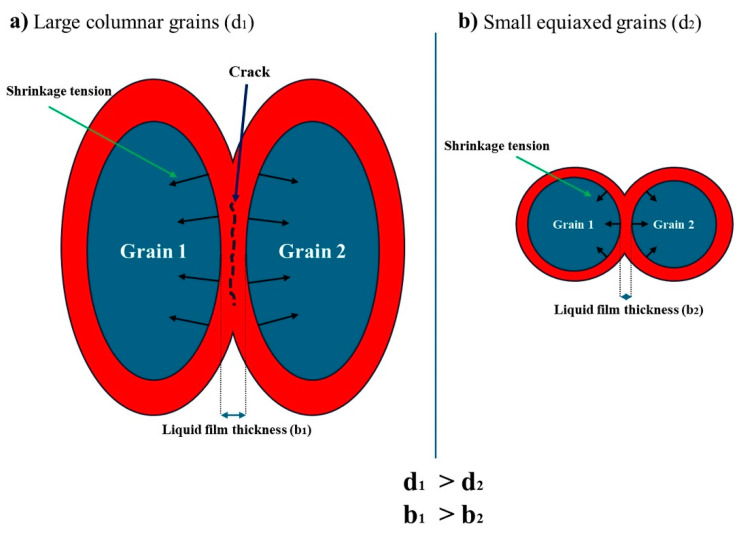
Schematic of the cracking mechanism in the final stages of solidification: (**a**) large columnar grains and (**b**) small equiaxed grains.

Inside the melt pools of the base and ZV alloys, the grains are almost columnar and their size is large (Figure 6c and Figure 7c). The addition of TiB_2_ particles shifts the grain growth from columnar to equiaxed (Figure 6, Figure 7 and Figure 8), significantly reducing the grain size in the B-3TiB and ZV-6TiB alloys. In terms of the effectiveness of grain refinement, the growth restriction factor (GRF) serves as a powerful parameter [40]. While Ti has the highest GRF, V and Zr also exhibit higher GRF values compared to Cu. The higher concentrations of Ti, Zr, and V in combination with higher TiB_2_ particles in the ZV-6TiB alloy effectively contribute to the significant grain size reduction after LSR relative to the B-3TiB alloy, as shown in Figure 6 and Figure 7. The main reason for crack mitigation is the grain structure modification. A schematic of the cracking mechanism in terms of grain structure and size is shown in Figure 14. In this illustration, d_1_, d_2_, and b_1_, b_2_ are the grain sizes and liquid film thicknesses in the final stages of solidification, respectively.

### 4.3. Advantage and Limitation

Al-Si-based AlSi10Mg alloys are the most commonly used aluminum alloys in the additive SLM process owing to their excellent processability and adaptability. In industrial applications involving complex-shaped materials, a stress-relieving treatment is often adopted because of the high residual stresses in the printed components. However, this treatment leads to a significant reduction in the mechanical properties, which necessitates a subsequent T6 heat treatment to restore the mechanical properties. Despite numerous efforts to optimize the heat treatments of conventional AlSi10Mg alloys, the mechanical properties after T6 treatment remain relatively low with yield strengths (YS) typically below 250 MPa [10].

A comparison of T6 mechanical properties between AlSi10Mg and the Al-Cu alloys in this study reveals a notable difference in mechanical performance. As shown in Table 2, the hardness of the B-3TiB and ZV-6TiB alloys reached 117–130 HV, whereas the SLMed AlSi10Mg alloys typically ranged from 60 to 100 HV. This indicates that the Al-Cu alloys in the present study surpass the T6 mechanical strength of conventional AlSi10Mg alloys by at least 15–30%. This improvement is most likely attributed to the fine grain strengthening and nanoscale θ′/θ″-precipitation strengthening, particularly in the ZV-6TiB alloy. The TiB-modified Al-Cu alloys in this study present a promising opportunity for new additive manufacturing materials that achieve a high mechanical performance while maintaining good processability.

High-strength Al-Cu alloys are generally susceptible to solidification cracks during rapid solidification [15]. The hot tearing susceptibility and microstructural evolution of Al-Cu 224 alloys that occur during rapid solidification were investigated using laser surface remelting. This study provided a scientific basis and practical method for developing Al-Cu alloys with good processability for rapid solidification processes, particularly the SLM process. The results revealed that the addition of appropriate amounts of TiB grain refiner could eliminate the solidification cracks and significantly improve the grain structure of Al-Cu alloys during the SLM process.

The approach used for developing TiB-modified alloys offers significant potential for the design and manufacture of high-strength Al-Cu alloys tailored in the SLM process. Al-Cu alloys typically require high energy density [47], a low scan speed, and low power during the SLM process, which increases production time and costs. The modifications presented in this study improve the processability of Al-Cu 224 alloys, enabling more efficient melting and solidification and thereby lowering both production time and costs. Such findings could pave the way for broader industrial adoptions of high-strength wrought aluminum alloys in SLM. Nevertheless, the implications and limitations of this study must be carefully considered. The enhancement of cracking resistance during rapid solidification with increasing TiB_2_ particle content in the cracking-prone Al-Cu alloys is undeniable. However, the use of laser-surface-melted tracks in the current approach did not consider the impacts of powder-based processing, thermal cycling and the overlapping of remelted tracks in subsequent layers, as in a typical SLM process. Therefore, the results herein provide only the first estimation of the attainable T6 strength and processing performance of the TiB-modified Al-Cu 224 alloys in a real SLM process. In this study, the laser surface remelting (LSR) process was used as a practical method to evaluate the grain refinement and mechanical strength of modified Al-Cu alloys. However, a comprehensive evaluation of materials and mechanical properties under real SLM production conditions remains a key limitation.

## 5. Conclusions

In the present study, the microstructural evolution, hot tearing susceptibility, and mechanical properties of five Al-Cu 224 alloys with and without the addition of TiB grain refiner were investigated using a laser surface remelting method. The following conclusions were drawn from the main findings:

(1) The addition of 0.3–0.65 wt% Ti to Al-Cu 224 alloys by Al-5Ti-1B grain refiner significantly reduced the hot tearing susceptibility during laser-based rapid solidification. With sufficient amounts of TiB grain refiner, solidification cracks could be completely eliminated in these Al-Cu alloys, making them promising high-strength alloys for the SLM process.
(2) The introduction of TiB_2_ particles into the melt pools from the TiB grain refiner changed the grain morphology from a coarse columnar to a fine equiaxed structure and significantly reduced the grain size. Following TiB modification, the grain sizes were reduced from 13 to 15 μm in the base alloys to 5.5 and 3.2 μm in the alloys with 0.3 wt% Ti (B-3TiB) and 0.65 wt% Ti (ZV-6TiB) additions, respectively.
(3) The modified B-3TiB and ZV-6TiB alloys reached hardness values of 117 and 130 HV after T6 heat treatment, which surpassed the values of conventional AlSi10Mg alloys by at least 15–30%. This was attributed to the strengthening contributions from fine equiaxed grains and from nanoscale θ′/θ″-precipitation.
(4) The laser surface remelting used in this study provides a simple and practical method to assess and optimize the processability and mechanical properties of high-strength aluminum alloys during laser-based rapid solidification processes. The processing and mechanical performances, and the microstructure–properties relationship of modified Al-Cu 224 alloys under real SLM process conditions, will be focused on in future studies.

## Figures and Tables

**Table 1:** Chemical compositions of experimental alloys (wt%).

	Si	Fe	Cu	Mn	Mg	Zr	V	Ti	Al
Base	0.06	0.12	4.7	0.35	0.14	0.01	0.01	**0.05**	Bal.
B-3TiB	0.22	0.12	4.59	0.36	0.10	0.01	0.02	**0.34**	Bal.
ZV	0.04	0.11	4.69	0.34	0.12	0.15	0.21	**0.05**	Bal.
ZV-3TiB	0.11	0.12	4.35	0.34	0.08	0.15	0.22	**0.31**	Bal.
ZV-6TiB	0.09	0.13	4.36	0.34	0.07	0.14	0.24	**0.65**	Bal.

**Table 2:** Comparison of mechanical properties between the current Al-Cu alloys and conventional SLMed AlSi10Mg alloys.

	Alloys	HV	Heat Treatment	References
1	B-3TiB	117	T6	Present study
2	ZV-6TiB	130	T6	Present study
3	AlSi10Mg	80 *	T6	[41]
4	AlSi10Mg	83	T6	[42]
5	AlSi10Mg	79	T6	[43]
6	AlSi10Mg	100	T6	[44]
7	AlSi10Mg	70 *	Stress relief	[45]
8	AlSi10Mg	88	Stress relief	[46]

* Converted from the yield stress, assuming that the yield stress is three times the hardness.

## Data Availability

The original contributions presented in this study are included in the article. Further inquiries can be directed to the corresponding author.
